# Ectopic Runx1 Expression Rescues Tal-1-Deficiency in the Generation of Primitive and Definitive Hematopoiesis

**DOI:** 10.1371/journal.pone.0070116

**Published:** 2013-07-29

**Authors:** Julia Tornack, Katharina Seiler, Andreas Grützkau, Joachim R. Grün, Masafumi Onodera, Fritz Melchers, Motokazu Tsuneto

**Affiliations:** 1 Max Planck Institute for Infection Biology, Berlin, Germany; 2 Institute for Stem Cell Biology & Regenerative Medicine, Stanford, Connecticut, United States of America; 3 Deutsches Rheumaforschungszentrum, Berlin, Germany; 4 National Research Institute for Child Health and Development, Tokyo, Japan; Hong Kong University of Science and Technology, China

## Abstract

The transcription factors SCL/Tal-1 and AML1/Runx1 control the generation of pluripotent hematopoietic stem cells (pHSC) and, thereby, primitive and definitive hematopoiesis, during embryonic development of the mouse from mesoderm. Thus, Runx1-deficient mice generate primitive, but not definitive hematopoiesis, while Tal-1-deficient mice are completely defective. Primitive as well as definitive hematopoiesis can be developed “in vitro” from embryonic stem cells (ESC). We show that wild type, as well as Tal-1^−/−^ and Runx1^−/−^ ESCs, induced to differentiation, all expand within 5 days to comparable numbers of Flk1^+^ mesodermal cells. While wild type ESCs further differentiate to primitive and definitive erythrocytes, to c-fms^+^Gr1^+^Mac1^+^ myeloid cells, and to B220^+^CD19^+^ B- and CD4^+^/CD8^+^ T-lymphoid cells, Runx1^−/−^ ESCs, as expected, only develop primitive erythrocytes, and Tal-1^−/−^ ESCs do not generate any hematopoietic cells. Retroviral transduction with Runx1 of Runx1^−/−^ ESCs, differentiated for 4 days to mesoderm, rescues definitive erythropoiesis, myelopoiesis and lymphopoiesis, though only with 1–10% of the efficiencies of wild type ESC hematopoiesis. Surprisingly, Tal-1^−/−^ ESCs can also be rescued at comparably low efficiencies to primitive and definitive erythropoiesis, and to myelopoiesis and lymphopoiesis by retroviral transduction with Runx1. These results suggest that Tal-1 expression is needed to express Runx1 in mesoderm, and that ectopic expression of Runx1 in mesoderm is sufficient to induce primitive as well as definitive hematopoiesis in the absence of Tal-1. Retroviral transduction of “in vitro” differentiating Tal-1^−/−^ and Runx1^−/−^ ESCs should be a useful experimental tool to probe selected genes for activities in the generation of hematopoietic progenitors “in vitro”, and to assess the potential transforming activities in hematopoiesis of mutant forms of Tal-1 and Runx1 from acute myeloid leukemia and related tumors.

## Introduction

In the mouse embryo the first hematopoietic cells develop extra-embryonically at day 7.5 of embryonic development (E7.5) in the yolk sac (YS) blood islands. There, a first wave of primitive hematopoiesis develops special types of myeloid cells as well as red blood cells that express fetal-type (ζ)-globin [1]. Thereafter, at E8.5–9.5, hematopoiesis is initiated at an intra-embryonic region known as the para-aortic splanchnopleura, which later contains the developing aorta, gonads and mesonephros, called the AGM-region [2]–[6]. The hematopoietic progenitors developing in YS and in AGM can be distinguished by the expression of AA4.1 (CD93) [7]. Red cells developing in this second wave of definitive hematopoiesis express adult-type (β)-globin. From E11.5 fetal liver is colonized by pluripotent hematopoietic stem cells (pHSCs) which develop red cells, myeloid cells and B1-type, CD5^+^ B-lymphocytes, while fetal thymus begins to generate γ/ð-TcR^+^ and α/β-TcR^+^ T-lymphocytes. From E13.5 pHSCs begin to participate in the development of bone and its marrow. There, they have the capacity to become long-term resting cells or, upon activation, to self-renew or differentiate into all the lineages of the hematopoietic cell system.

The transcription factors SCL/Tal-1 (Stem cell leukemia/T cell acute leukemia 1) [8] and AML1/Runx1 (Acute myeloid leukemia 1/Runt related transcription factor 1) [9]–[10] are master regulators for both YS- and AGM-derived hematopoiesis. During embryonic development, Tal-1 is expressed in intra- and extra-embryonic mesoderm at day E7.5, in the YS blood island at E8.5, and thereafter in adult hematopoietic tissues. Tal-1^−/−^ mice die at E9.5 due to a failure to generate any hematopoietic progenitors, because development is arrested at a hemangioblast-like blast-colony-forming stage, that is unable to generate the normal endothelial and hematopoietic progeny, i.e. pHSCs and all the blood cell lineages [8], [11]–[13]. However, once pHSCs have been formed, Tal-1 becomes dispensable for the continued life-long functions of pHSCs, i.e. for engraftment after transplantation, self-renewal, long-term repopulating potency and multipotent differentiation into myeloid and lymphoid lineages, while proper development to erythroid and megakaryocytic cells remains dependent on Tal-1 expression [14].

Downstream of Tal-1, Runx1 is involved in the onset of the definitive hematopoietic program. In fact, Tal-1 directly controls the expression of Runx1 [15]–[17]. Runx1 is first seen expressed at E7.5 in extra-embryonic mesodermal cells and then transiently in primitive erythrocytes. In AGM, Runx1 expression is detected at E10.5, i.e. at the time when the first hematopoietic stem cells develop [18], [19]. Runx1^−/−^ mice are able to initiate YS-derived hematopoiesis but then die in utero at E12.5 [10], [20]. At that time, fetal liver contains only primitive erythroblasts. Runx1^−/−^ embryos show a complete block in the establishment of the definitive hematopoietic program, as definitive erythroid, myeloid and lymphoid cells are absent [10]. Restoration of Runx1 expression in Runx1-reversible knock-out mice, in the Tie2^+^ cell compartment during embryogenesis rescues the generation of clonogenic hematopoietic progenitors and the differentiation of the fetal phases of lymphoid and myeloid cell development [21].

The different primitive and definitive, embryonic and adult lineages of erythroid cells, myeloid cells and lymphocytes can be developed in “in vitro” cultures from embryonic stem cells (ESC) and from induced pluripotent stem cells (iPS) [22], [23]. Therefore, the ability of ESCs to generate hematopoietic progenitors using an established “in vitro” culture system provides a good approach for studying the functions of transcription factors that probably play critical roles in the earliest events of hematopoietic development. However, until today it has not been possible to generate long-term reconstituting pluripotent hematopoietic stem cells from such differentiating ESCs [23]. Here, we have attempted to define the potential function of Runx1 downstream of Tal-1 in the development of primitive as well as definitive hematopoietic progenitors and their lineage specification thereafter. We reconstitute a Tal-1**^−/−^** ESC line for Runx1 expression by transduction with a retroviral vector. As a control, we also reconstitute with the same retroviral vector a Runx1^−/−^ ESC line. We utilize different ”in vitro” culture systems that allow us to compare the potential of wild type (WT), Tal-1^−/−^ and Runx1^−/−^ ESCs, as well as Runx1^−/−^ or Tal-1^−/−^ ESCs reconstituted with Runx1 to develop to erythroid, myeloid and lymphoid cells. Surprisingly, ectopic expression of retrovirally transduced Runx1 in Tal-1^−/−^ cells is sufficient to inhibit apoptosis of ESC-derived, differentiating Flk1^+^ progenitors, allows the development of CD45^+^ hematopoietic progenitors, and rescues primitive as well as definitive erythropoiesis, myelopoiesis and lymphopoiesis. Compared with WT ESCs this reconstitution of myelopoietic and lymphopoietic developments occurs with low efficiencies, although both Runx1-transduced Tal-1^−/−^ and Runx1^−/−^ ESCs do it comparably well, while erythropoietic developments are less efficient. Our finding that ectopic expression of Runx1 rescues all of primitive and definitive hematopoiesis in Tal-1^−/−^ cells demonstrates that, in the transcriptional hierarchy of the earliest events of hematopoietic development, Tal-1 is necessary to initiate Runx1 expression that, thereby, facilitates the proper hematopoietic development from uncommitted mesoderm.

## Materials and Methods

### Cell Lines

The OP9 and OP9-DL1 stromal cell lines [24], [25] (a kind gift of Dr. Zuniga-Pfluecker, University of Toronto) were cultured in alpha-minimum essential medium (αMEM, Gibco-Invitrogen) supplemented with 20% FCS (Sigma-Aldrich). The bone marrow–derived stromal cell line, ST2 [26], was maintained in DMEM (Gibco-Invitrogen) supplemented with 10% FCS (Gibco-Invitrogen). ESC lines wild type J1 [11], Runx1^−/−^ J1 (a kind gift of Dr. Nancy A. Speck, Abramson Family Cancer Research Institute) [10] and Tal-1^−/−^ J1 (a kind gift of Stuart H. Orkin, MD, Harvard Stem Cell Institute) [11] were maintained on irradiated mouse embryonic fibroblasts (MEFs) in Dulbecco’s modified essential medium (DMEM GlutaMAX™, Gibco-Invitrogen) supplemented with 15% heat-inactivated FCS (Gibco-Invitrogen), 10^−4^ M 2-Mercaptoethanol (Sigma-Aldrich), 1×nonessential amino acids (Gibco-Invitrogen), 1×sodium pyruvate (Gibco-Invitrogen), and leukemia inhibitory factor (LIF) equivalent to 1.000 U/ml on 0.1% gelatin-coated culture dishes. The Platinum-E (Plat-E) vector packaging cell line [27] was cultivated in DMEM (Gibco-Invitrogen) supplemented with 10% FCS (Gibco-Invitrogen). Cytokine supernatants were produced by using the appropriate hybridoma cell lines: IL-7 (J558L/IL-7) [28], SCF (CHO-SCF, a kind gift of Dr. Thorsten Feyerabend, Universität Ulm) [29], Flt-3L (Sp2.0-Flt3-L, a kind gift of Dr. Paulo Vieira, Institute Pasteur, Paris) [30], LIF (J558-LIF) [23].

### Retroviral Vectors, Production of Retroviral Particles, and Transduction of Target Cells

The retroviral vector expressing Runx1 were composed of a mouse Runx1 cDNA sequence followed by an internal ribosomal entry site (IRES) and a red fluorescent protein (humanized Kusabira-Orange, huKO) (pGCDNsam-Runx1-IRES-huKO). The empty vector control were composed of an internal ribosomal entry site (IRES) and a red fluorescent protein (pGCDNsam-IRES-huKO). Vector particles were produced by transient transfection of Plat-E packaging cells. Cells were seeded at 3×10^5^ cells/ml in six-well-plates. After 24 h, the cells were transfected with 1 mg retroviral vector plasmid mixed with 5 µl Lipofectamine™ (Invitrogen) per 3×10^5^ cells for 5 h. The cell culture supernatants were changed after 24 h and harvested after further 48 h. For the transduction, Tal-1^−/−^ J1 ESCs or Runx1^−/−^ J1 ESCs were differentiated for four days “in vitro”. 3×10^5^ cells of these differentiating Tal-1^−/−^ J1 ESCs were spin-infected with 100 µl virus supernatant, 900 µl αMEM and Protamine sulfate salt (Sigma, P4505) at 2400 g for 2 h 30 min at 30°C. Afterwards, differentiation cultures were continued on a layer of OP9.

### Differentiation of ES Cells

Undifferentiated ESCs were plated at 1.25×10^3^ cells/ml in six-well plates (Corning) on pre-seeded confluent, non-irradiated OP9 stromal cells and cultured in αMEM supplemented with 20% FCS (Gibco-Invitrogen). On day 5, cells were harvested by trypsinization and reseeded at 5.5×10^4^ cells/ml per six-well plate (Corning) onto confluent non-irradiated OP9 stromal cells in αMEM/20% FCS. For the induction of B cell development 3% stem cell factor-conditioned medium (SCF-CM) and 2% Flt3-L-CM were added to cultures of semi-confluent, non-irradiated OP9 stromal cells for the next 7 days. Afterwards, at day 12 of culture the media was changed to Iscove’s modified Dulbecco’s medium (IMDM)/2% FCS/0.03% Primatone/1% IL-7-CM/2% Flt3-L-CM on non-irradiated OP9 stromal cells. On day 15, all cells were suspended as single cells, filtered over nylon wool, and subsequently passed to fresh semi-confluent OP9 stromal cells. On day 21, cells were harvested and analyzed. T cell development was induced on a layer of semi-confluent, non-irradiated OP9-DL1 stromal cells in IMDM/2% FCS/0.03% Primatone/0.5% IL-7-CM/2% Flt3-L-CM. Osteoclasts were developed by co-culture on irradiated ST2 stromal cells from differentiating ESCs at day 10 in αMEM/10% FCS with the addition of 10^−8^ M 1α,25-dihydroxyvitamin D3 (VitD_3_; Biomol Research Laboratories) and 10^−7^ M dexamethasone (Dex; Sigma Chemical Corp.). They could be detected on day 16 by their expression of tartrate-resistant acid phosphatase (TRAP). Primitive as well as definitive erythrocytes were induced from differentiating ESCs at day 5 on fresh confluent, non-irradiated OP9 stromal cells in αMEM/20% FCS/3% SCF-CM/0.3% recombinant human erythropoietin (rhEpo, R & D Systems). On day 8 or day 21, cells were prepared for either FACS analyses or real-time quantitative reverse transcription–polymerase chain reaction analyses (qRT-PCR). For the induction of myeloid cell development (macrophages, granulocytes), from day 10 on cells were cultured on confluent, non-irradiated OP9 stromal cells in αMEM/20% FCS/3% SCF-CM until day 21. Differentiated myeloid or erythroid cells were enriched with FACS and cytospins were performed. The cytospin samples were airdried, and May-Grünwald-Giemsa-stainings were performed.

### Antibodies and Flow Cytometric Analysis

Cy5 anti-mouse c-Kit (2B8), PE-Cy7 anti-mouse CD45 (30-F11), PE anti-mouse AA4.1 (AA4.1), PerCpCy5.5 anti-mouse Ter119 (TER-119), PE anti-mouse CD71 (R17217), PB anti-mouse Mac1 (M1/70), Cy5 anti-mouse CD19 (1D3), PE-Cy7 anti-mouse B220 (RA3-6B2), biotin anti-mouse Tie2 (TEK4), PE anti-mouse Flk1 (Avas12A1), PE anti-mouse Gr1 (RB6-8C5), PB anti-mouse CD44 (IM7), PerCpCy5.5 anti-mouse CD25 (PC61), PE-Cy7 anti-mouse CD4 (GK1.5), Cy5 anti-mouse CD8 (53-6.7), Cy5 Annexin V and APC-coupled Streptavidin were obtained from eBioscience, San Diego, CA.

Single cell suspensions from the cell culture were prepared by filtration over nylon wool and washing with ice-cold FACS buffer (PBS/2% FCS). Cells were incubated with heat-inactivated rabbit serum for 10 min followed by staining with a combination of conjugated antibodies in FACS buffer for 30 min, propidium iodide (PI, Calbiochem) diluted in FACS buffer for 10 min and finally washed with FACS buffer. Cells were FACS-analysed on an LSRII flow cytometer (BD Biosciences). To analyze surface marker expression dead cells were discriminated by PI staining. For the Cy5 Annexin V staining, 7-Amino-Actinomycin (7-AAD) were used as a vital dye to identify early apoptotic cells (7-AAD negative, Cy5 Annexin V positive) and cells that have died already (7-AAD positive, Cy5 Annexin V positive).

### RNA Isolation, Microarray Hybridization, and Chip Data Analysis

Gene-expression profiling of three biological replicates was performed at days 4, 5, and 6 during the differentiation process of WT J1 ESCs (9 samples), and at day 6 during the differentiation process of either Runx1^−/−^ J1 or Tal-1^−/−^ J1 ESCs (3 samples each). Total RNA was extracted using the RNeasy Mini kit (Qiagen). The integrity and amount of isolated RNA was assessed for each sample using an Agilent 2100 Bioanalyzer (Agilent, Waldbronn, Germany) and a NanoDrop ND-1000 spectrophotometer (NanoDrop Technologies, Wilmington, DE). Complementary DNA was synthesized from 3–5 µg total RNA, using reagents recommended in the technical manual *GeneChip Expression Analysis* (Affymetrix, Santa Clara, CA). The in vitro transcription, necessary for the synthesis of biotinylated complementary RNA (cRNA) was performed using the Enzo RNA Transcript Labeling kit (Affymetrix). Fifteen micrograms of fragmented cRNA of each sample were hybridized to nine Mouse Genome 430-2 arrays (Affymetrix). Hybridization was performed in a Hybridization Oven 640, and chips were washed and stained in the Fluidics Station 400 (both Affymetrix), according to procedure 2 as described in the technical manual. Finally, the arrays were scanned with a GeneChip Scanner 3000 using the GCOS software, both Affymetrix. All relevant GCOS data of quality checked microarrays were analyzed with High Performance Chip Data Analysis (HPCDA, unpublished), using the BioRetis database (www.bioretis-analysis.de), as described and validated previously [31]. Used query parameters for database filtering process was described earlier several times [32]. For hierarchical cluster analysis, we used the program Genes@Work [33] with gene vectors for normalization and Pearson w/mean for similarity measure. As cluster type, we used center of mass. All chip data were uploaded to GEO (accession number is: GSE46970) and publicly available.

### Real-time Quantitative Reverse Transcription–polymerase Chain Reaction Analyses (qRT-PCR)

Total RNA was purified from differentiating ESCs using Trizol (Biozol) and 1 µg of total RNA was reverse-transcribed by SuperScriptIII (Invitrogen) primed with oligodT. Expression of mRNAs was quantitatively assessed by quantitative real-time PCR using the QuantiTect SYBR Green PCR Kit in a 7900HT Fast Real-Time PCR system with the GAPDH gene as reference. For each reaction (25 µL final volume), 5 µl of cDNA sample (50–100 ng/reaction) were mixed with 0.5 µl of primer pairs (400 nM final), 10 µL of SYBR Green mix and 9 µl RNase-free H_2_O. Each sample was assayed in triplicate for every run. Control RNA from wild type cells or an indicated specific control was used to construct a standard curve for all inspected genes, proving specificity and reliability of the designed oligonucleotide pairs. The following primers were used:

Tal-1: 5′-ATAGCCTTAGCCAGCCGCTC-3′ and 5′-GCCGCACTACTTTGGTGTGA-3′, Runx1∶5′-GCCTCCTTGAACCACTCCAC-3′ and 5′-GTTCTGCAGAGAGGCTGGTC-3′, PU.1: 5′ -TGCGGAGAAATCCCAGTAGT-3′ and 5′ -CTCCATCGGATGACTTGGTT-3′, c-fms: 5′-GGGCAACTGAGTAGGGTCAA-3′ and 5′-GTGCTACTGCTGTTGCTGCT-3′, GATA1: 5′-CAGGGCAGAATCCACAAACT-3′ and 5′-TCCTCTGCATCAACAAGCC-3′, Klf1: 5′-GACGATGTCCAGTGTGCTTC-3′ and 5′-CTACTCCAAGAGCTCGCACC-3′, Nfe2: 5′-CAGGTCTCCACAAGCACAAA-3′ and 5′-AGCTCTGACCCAGCCTCTC-3′, Rag1: 5′-GCAGAACTGAAGCTCAGGGT-3′ and 5′-TTGCTATCTCTGTGGCATCG-3′, Ikzf1: 5′CCTTTCTGGGTAAAGGAGGC–3′ and 5′-GACACTCCAGATGAAGGGGA-3′, GAPDH: 5′-CGTCCCGTAGACAAAATGGT-3′ and 5′-TTGATGGCAACAATCTCCAC-3′, ζ-globin: 5′-GCTCAGGCCGAGCCCATTGG-3′ and 5′-TAGCGGTACTTCTCAGTCAG-3′, β-globin: 5′-CACAACCCCAGAAACAGACA-3′ and 5′-CTGACAGATGCTCTCTTGGG-3′, EBF1: 5′-GACACCACGACCTGGAATCT-3′ and 5′-CCGAAATGAGACTCCCTCAG-3′, VpreB: 5′-TGCCAATGTTATGGTCGTTG-3′ and 5′-ATGCTGCTGGCCTATCTCAC-3′, Igα: 5′-CATGTCCACCCCAAACTTCT-3′ and 5′-GGTACCAAGAACCGCATCAT-3′, CD3: 5′-TCGTCACTGTCTAGAGGGCA-3′ and 5′-CCTCCTAGCTGTTGGCACTT-3′, preTα: 5′-TCTACCAGCAGTGTGATGGG-3′ and 5′-GGTCATGCTTCTCCACGAGT-3′.

The cycling program included initial hold for 15 min at 95°C. Each PCR was performed at 45 cycles consisting of 20 s at 95°C melting, 40 s at 60°C annealing, and 40 s at 72°C elongation. A threshold cycle value (Ct) was chosen to fall within the linear phase for each set of curves. Triplicate wells were averaged, and target gene values were normalized to GAPDH expression levels.

### Reverse Transcription–polymerase Chain Reaction Analyses (RT-PCR)

Gene expression was analyzed by RT-PCR using the following primers: Runx1 exogenous: 5′-CGGTTCCTACCAGTTCTCCA-3′ and 5′-CGGAATTCGTTAACCTCGAT-3′, Runx1 endogenous: 5′-GAGGGCAGCCATAGCAACT-3′ and 5′-AGTTTCCCTCCGGGATTCTT-3′, HPRT: 5′-AGTTCTTTGCTGACCTGCTG-3′ and 5′-GCTTTGTATTTGGCTTTTCC-3′. One µg cDNA was amplified in 1×PCR buffer with KCl supplemented with 0.2 mM dNTPMix, 1.5 mM MgCl2, 1 µM of each primer and 5 U Taq DNA-Polymerase in a total volume of 20 µl.

### TRAP-staining

WT, Runx1^−/−^, Tal-1^−/−^, and Runx1-reconstituted Tal-1^−/−^ or Runx1^−/−^ cells that were cultivated under osteoclast differentiation conditions were fixed with 10% formaldehyde (Roth) for 10 minutes at room temperature and with ethanol/acetone (50∶50 v/v; Roth) for 1 minute. Thereafter, the cells were washed with deionized water and stained with fast red violet LB-salt (Sigma) mixed with tartrate resistant acid phosphatase (TRAP) solution containing 59.3 M of sodium tartrate (Sigma-Aldrich), 165.7 M sodium acetate (Sigma-Aldrich), 0.56 mg/ml naphthol AS-MX phosphate (Sigma) for 5 min at room temperature. Under a microscope TRAP-positive cells were visible as red-stained cells.

### Statistical Analysis

For all statistical analyses, PRISM (Version 5, GraphPad, San Diego) software was used. Dispersion is presented as the SD, unless stated otherwise.

## Results

### Developmental Potential of WT, Runx1^−/−^ and Tal-1^−/−^ ES Cells (ESC)

**Figure 1:**
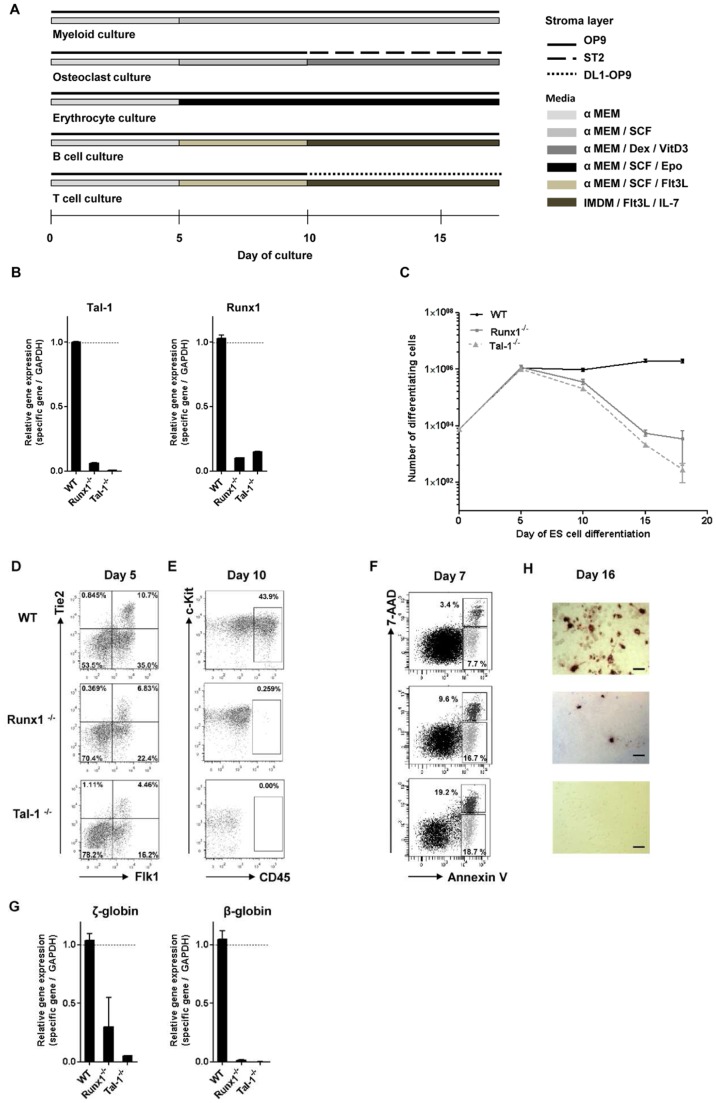
Comparison of the developmental potential of WT, Runx1^−/−^, and Tal-1^−/−^ ESCs. (A) Undifferentiated ESCs were seeded on OP9 stromal cells (day 0). From day 5 of the culture, the conditions for the different lineages are the same. From day 5 on, different cytokines were added to the cultures and feeding stromal cell layers were changed according to the lineage. (B) qRT–PCR analyses were performed from 8 days differentiated (αMEM/3% SCF) WT, Runx1^−/−^, and Tal-1^−/−^ ESCs out of 3 independent experiments. Bars represent mean ± SD. (C) Representative growth curves of WT ESCs in comparison to Runx1^−/−^ and Tal-1^−/−^ ESCs from day 0–18 of the culture (n = 3). Dots show mean ± SD. Development of (D) mesodermal cells, (E) hematopoietic progenitors, (G) erythrocytes and (H) osteoclasts that are differentiated from undifferentiated cells. FACS blots are representative examples of differentiated ESCs. Osteoclasts were identified by staining with TRAP-solution. (F) Apoptosis was evaluated after staining with Annexin-V at day7 of the culture. Flow cytometry profile represents Annexin-V-Cy5 staining in *x* axis and 7-AAD in *y* axis. OP9: stromal cell line, DL1: Delta-like 1, ST2: stromal cell line, αMEM: alpha-minimum essential medium, IMDM: iscove’s modified dulbecco’s medium, SCF: stem cell factor, Dex: dexamethasone, VitD_3_: Vitamin D_3_, Epo: erythropoietin, IL: interleukin, Flt3-L: Fms-like tyrosine kinase 3 ligand; SD: standard deviation.

The ability of ESCs to generate hematopoietic progenitors “in vitro” can be used for studies of the function of transcription factors that are expected to play roles in primitive as well as definitive hematopoiesis. To investigate the role of Runx1 in hematopoietic development, we first compared the “in vitro” development of WT ESCs with that of Tal-1**^−/−^** ESCs and Runx1**^−/−^** ESCs to erythroid, myeloid and lymphoid cells (Fig. 1 A). The absence of either Tal-1 or Runx1 expression in differentiating Tal-1**^−/−^** or Runx1^−/−^ ESCs was confirmed by qRT-PCR (Fig. 1 B). Within the first 5 days of culture WT, Tal-1**^−/−^** and Runx1**^−/−^** ESCs all developed 100 fold increased numbers of cells (Fig. 1 C), of which 20–40% were found to be Flk1^+^/CD41^−/^CD45^−^ mesodermal progenitors of hematopoietic cells (Fig. 1 D). Within the next 5 days, WT ESCs further differentiated in SCF-containing medium first to CD41^+^ (data not shown) and then to Flk-1^−/^CD41^−/^CD45^+^ hematopoietic cells with increasing numbers (Fig. 1 C and E). By contrast, differentiating Runx1**^−/−^** ESCs did not increase, but decreased to half the numbers of cells after day 5, although low numbers of CD41^−/^CD45^+^ hematopoietic cells developed (Fig. 1 E). It is possible that the CD41^−/^CD45^+^ cells in Runx1-deficient ESC cultures are primitive hematopoietic cells [34], because this type of hematopoiesis is observed in Runx1^−/−^ YS [10], and YS hematopoietic cells produce CD45^+^ cells in culture [35]. The decrease in cell numbers was even more pronounced in cultures of differentiating Tal-1**^−/−^** ESCs (Fig. 1 C), where CD45^+^ hematopoietic cells remained below the level of detection (Fig. 1 E). In fact, Tal-1**^−/−^** and Runx1**^−/−^** cells died by apoptosis (Fig. 1 F).

From Runx1**^−/−^** ESCs, but not from Tal-1**^−/−^** ESCs low numbers of primitive erythrocytes expressing fetal (ζ) but not adult (β) type-globin developed (Fig. 1 G), and low numbers of TRAP^+^ osteoclasts were induced (Fig. 1 H). Progenitors of myeloid and lymphoid cells were induced in culture between day 5 and 10 from WT ESCs, but not from Runx1**^−/−^** ESCs and Tal-1^−/−^ ESCs (data shown below). In confirmation with earlier findings, our data show that Tal-1^−/−^ ESCs could not be differentiated into any hematopoietic progenitors (Fig. 1 E), and that, therefore, neither primitive (e.g. primitive erythrocytes) nor definitive hematopoietic cell lineages (e.g. definitive erythrocytes, myeloid cells, and lymphoid cells; data shown below) could be developed in the culture. As expected, Runx1**^−/−^** ESCs developed cells of primitive hematopoiesis, e.g. primitive erythrocytes expressing fetal (ζ) but not adult (β) type-globin (Fig. 1 G). Additionally, low numbers of CD41^−/^CD45^+^ cells and TRAP^+^ osteoclasts were developed (Fig. 1 E and H).

### Microarray Analysis Identifies Hematopoietic- and Erythroid-related Genes being Downregulated in Tal-1-deficient and Runx-1-deficient Differentiating ESCs

**Figure 2:**
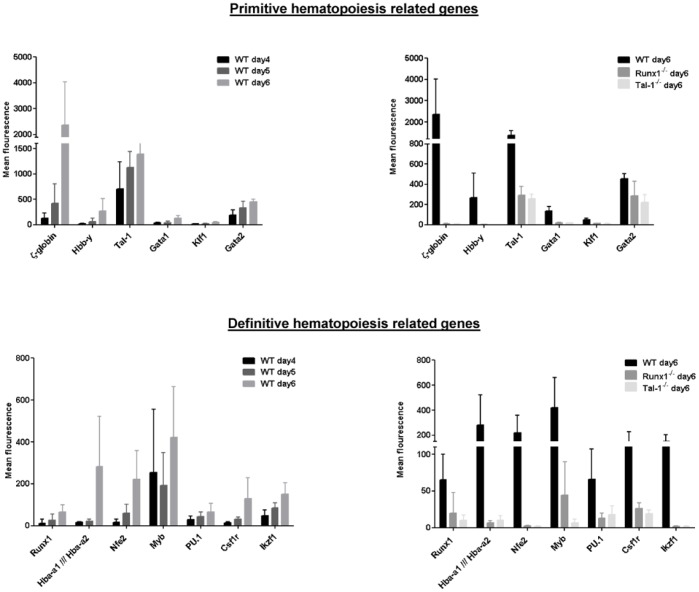
Microarray analysis of differentiated WT, Runx1^−/−^ and Tal-1^−/−^ ESCs. Undifferentiated WT ESCs were differentiated until day 4, 5, and 6, Runx1^−/−^ and Tal-1^−/−^ ESCs were differentiated until day 6 of the culture. Flk1^+^ cells were purified by flow cytometry. Three biologic replicates were performed for each time point of each cell line. Bars represent the relative expression of selected genes measured by the use of Mouse Genome 430-2 arrays (Affymetrix). Bars represent mean ± SD. SD: standard deviation.

In order to identify genes that are activated in differentiating WT ESCs, but are missing in Tal-1**^−/−^** and Runx1**^−/−^** ESCs, and which might be involved in the generation of definitive hematopoietic progenitors and their specification thereafter, we performed microarray analyses on purified Flk-1^+^ cells, differentiated from WT ESCs for 4, 5, and 6 days, as well as from Runx1**^−/−^** and Tal-1^−/−^ ESCs differentiated for 6 days “in vitro”. A selected set of suggested primitive (e.g. Tal-1, GATA1, GATA2, Klf1, and fetal (ζ) type-globin) as well as definitive hematopoiesis-related genes (e.g. Runx1, c-Myb, Nfe2, PU.1, c-fms, and Ikzf1) are differentially regulated for 4, 5, and 6 days differentiated WT ESCs as well as for 6 days differentiated Tal-1**^−/−^** and Runx1**^−/−^** ESCs in comparison to WT ESCs (Fig. 2). Subsequently, these microarray expression analyses were validated by qRT-PCR analyses (data shown below). These analyses confirm and complement many of the results of earlier genome-wide expression analyses [16], [17], [36], showing that expressions of all of the selected hematopoietic- and erythroid-related genes, including Runx1, increase in differentiating WT ESCs, while they are totally missing under Tal-1^−/−^ conditions (Fig. 2). In addition, many of these genes, by comparison with WT ESCs, were reduced in differentiating Runx1^−/−^ ESCs (Fig. 2). These analyses support earlier conclusions by others, that Runx1 acts downstream of Tal-1 in transcriptional controls of hematopoiesis [15]–[17].

### Retroviral Transduction of Tal-1^−/−^ ESCs and Runx1^−/−^ ESCs with Transgenic Runx1

**Figure 3:**
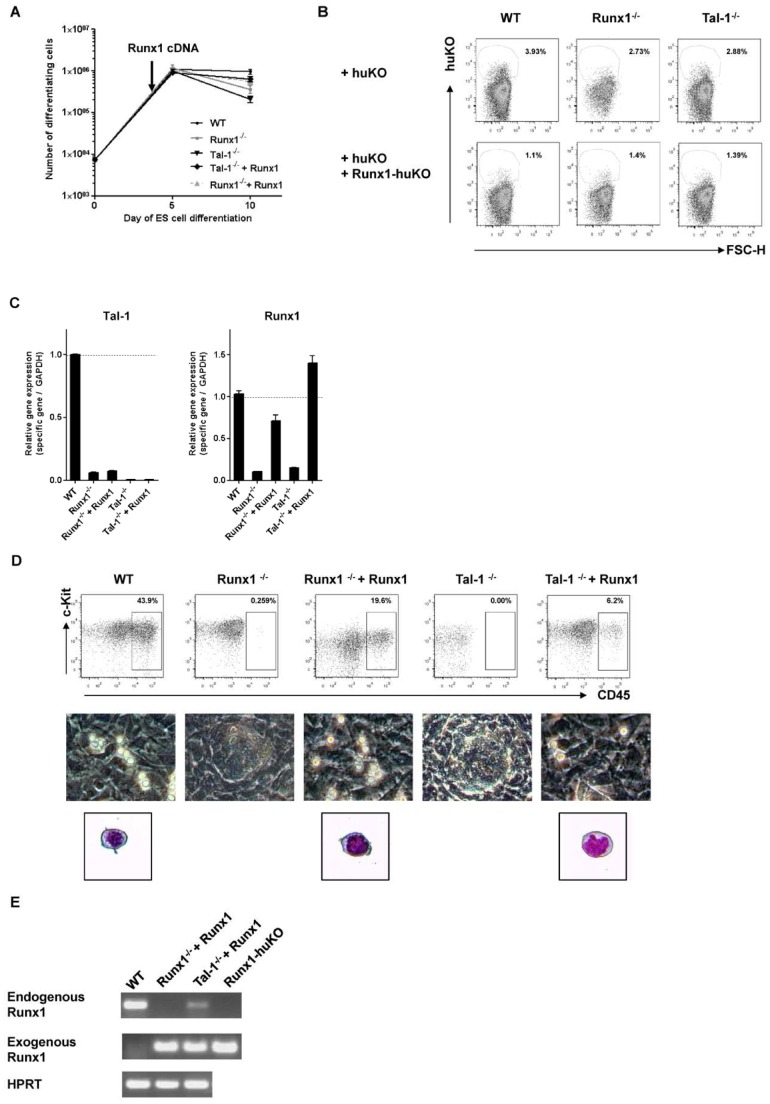
Development of CD45^+^ hematopoietic progenitors from Runx1-expressing Tal-1^−/−^ and Runx1^−/−^ ESCs. (A) Runx1^−/−^ and Tal-1^−/−^ ESCs, that were differentiated until day 4 of the culture, were transduced (indicated with an arrow) with a retroviral vector containing Runx1 cDNA (pGCDNsam-Runx1-IRES-huKO). Representative growth curves of differentiating ESCs from day 0–10 of the culture (n = 3). Dots show mean ± SD. (B) Determination of the transduction efficiency by single transductions of WT, Tal-1^−/−^ ESCs and Runx1^−/−^ ESCs with the empty vector alone (pGCDNsam-IRES-huKO), or together with the Runx1-containing vector (pGCDNsam-Runx1-IRES-huKO). (C) Runx1 as well as Tal-1 expression was analyzed 6 days later by qRT-PCR. (D) Development of CD45^+^ hematopoietic progenitors that are differentiated from undifferentiated ESCs for 10 days. FACS blots are representative examples of differentiated ESCs. Morphology of differentiated ESCs was examined by light microscopy at day 10 of the culture and by May-Grünwald-Giemsa-staining. (E) CD45^+^ cells were FACS-purified with the intention to distinguish the expression of endogenous and exogenous expression of Runx1 in differentiated cells. SD: standard deviation, IRES: internal ribosomal entry site, huKO: humanized Kusabira-Orange, OP9: stromal cell line.

Since Tal-1 induces Runx1 expression, and since Runx1 expression induces definitive hematopoiesis we reasoned that transgenic expression of Runx1, introduced by retroviral transduction into differentiating Tal-1^−/−^ ESCs, might rescue some of the molecular and cellular features of definitive hematopoiesis, as they can develop from WT ESCs “in vitro”. Therefore, we transduced Flk1^+^ Tal-1**^−/−^** ESCs differentiated for 4 days “in vitro” with a retroviral vector containing the cDNA form of Runx1 (Fig. 3 A), followed by an internal ribosomal entry site (IRES) and a red fluorescent protein (humanized Kusabira-Orange, huKO) (pGCDNsam-Runx1-IRES- huKO, see also Materials and Methods). In parallel, to test for the efficiency of Runx1 transduction in a possible subsequent rescue of molecular and cellular features of definitive hematopoiesis we also transduced 4 days-differentiated Runx1^−/−^ ESCs with the retroviral, Runx1-containing vector.

We determined the transduction efficiency of the Runx1-containing retroviral vector by single transductions of WT, Tal-1^−/−^ and Runx1^−/−^ ESCs with the empty vector alone (pGCDNsam-IRES-huKO), or together with the Runx1-containing vector (pGCDNsam-Runx1-IRES-huKO), since we were not able to detect red fluorescent protein expression when the cells were transduced with the Runx1-containing vector alone. We think that this is the consequence of the IRES element in the vector that does not work in differentiating ESCs, as it is known that IRES works in different ways in different type of cells [37], [38]. Two days after transduction (day 6), 2–4% of the single-transduced cells (WT: 1–3×10^4^ of total cells, Runx1^−/−^ : 0.8–1.6×10^4^ of total cells, Tal-1^−/−^ : 0.76–1.5×10^4^ of total cells) were detected as red fluorescent cells (Fig. 3 B). On the other hand, 1–1.5% (WT: 0.64–0.96×10^4^ of total cells, Runx1^−/−^ : 0.4–0.6×10^4^ of total cells, Tal-1^−/−^ : 0.36–0.52×10^4^ of total cells) of the double-transduced cells were detected expressing red fluorescence after transduction (Fig. 3 B). This 2-fold reduction of the transduction efficiency is expected, if both vectors transfect and transduce the cells equally efficiently. This implies that the transduction efficiency of the vector carrying the Runx1 gene is between 2 and 4%. In addition, to confirm the expression of Runx1 in the transduced Tal-1^−/−^ and Runx1^−/−^ cells we analyzed the Runx1 expression 6 days later by qRT-PCR (Fig. 3 C). As expected, in the transduced Runx1^−/−^ cells Runx1 mRNA was expressed, though at lower levels than in WT ESCs differentiated for 10 days “in vitro”. In the transduced Tal-1^−/−^ cells Runx1 was detected at higher levels than in WT cells (Fig. 3 C).

### Development of CD45^+^ Hematopoietic Progenitors from Runx1-expressing Tal-1^−/−^ ESCs

Within the first 5 days of culture WT, Tal-1**^−/−^**, Runx1**-**expressing Tal-1**^−/−^**, Runx1**^−/−^**, and Runx1**-**expressing Runx1**^−/−^** ESCs developed 100-fold increased numbers of cells (Fig. 3 A), of which 20–40% were Flk1^+^ mesodermal progenitors of hematopoietic cells (shown for the non-transduced cells in Fig. 1 D). The Runx1-transduced cells continued to express Flk1 (data not shown). After transfer to SCF-containing media at day 5, WT, Runx1-transduced Runx1**^−/−^**and Runx1-transduced Tal-1**^−/−^** ESCs, but not Tal-1**^−/−^** ESCs differentiated to morphologically identifiable CD45^+^ hematopoietic progenitors at days 10 to 12 of culture (Fig. 3 D). In comparison with WT-differentiating cells Runx1^−/−^ cells, Runx1-transduced Runx1 cells and Runx1-transduced Tal-1**^−/−^** cells all developed CD45^+^ cells, though in lower numbers (Fig. 3 D). Compared with WT ESCs differentiated CD45^+^ cells, set as 100%, in this representative experiment Runx1**^−/−^** cells yielded only 0.5%, Runx1-transduced Runx1^−/−^ cells only 40%, and Runx1-transduced Tal-1**^−/−^** cells only 14% CD45^+^ cells (Fig. 3 D). CD45^+^ cells on day 10 of differentiation were FACS-purified with the intention to distinguish endogenous from exogenous Runx1 expression in differentiated cells (Fig. 3 E). Thereby, we detected endogenous Runx1 expression in differentiated WT ESCs, but not in purified populations of CD45^+^ cells from Runx1-transduced Runx1^−/−^ cells. However, in Runx1-transduced Tal-1^−/−^ cells low levels of endogenous Runx1 expression was detected (Fig. 3 E). Exogenous Runx1 expression was only detected in sorted CD45^+^ cells of Runx1-transduced Runx1^−/−^ and Tal-1^−/−^ cells, but not in differentiated WT cells. The vector itself was used as control to confirm that the PCR detected the exogenous Runx1 cDNA (Fig. 3 E).

We conclude that not only ectopic expression of Runx1 in Runx1**^−/−^** cells, but, surprisingly, also ectopic expression of Runx1 in Tal-1**^−/−^** ESCs rescued the Tal-1 defect in the generation of CD45^+^ hematopoietic progenitors. Therefore, we next probed the functions of these progenitors to develop further along erythroid, myeloid and lymphoid lineages “in vitro”.

### Myelopoiesis from Runx1-expressing Tal-1^−/−^ ESCs

**Figure 4:**
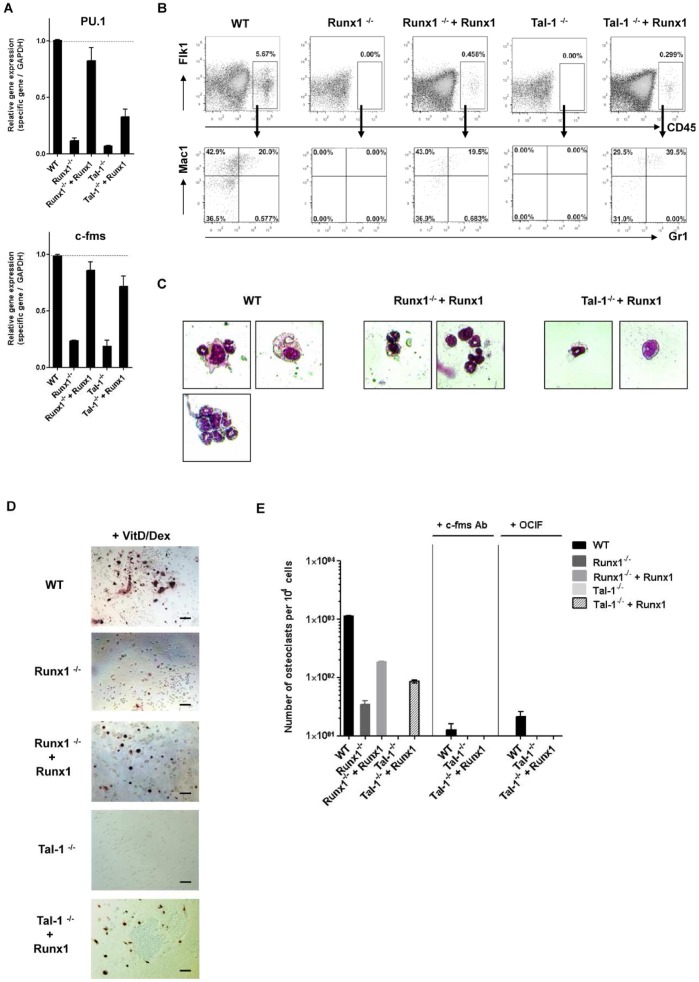
Development of myeloid cells from Runx1-expressing Tal-1^−/−^ and Runx1^−/−^ ESCs. (A) qRT–PCR analyses were performed from 8 days differentiated ESCs on day 8 of the culture out of 3 independent experiments. Bars represent mean ± SD. (B) Development of CD45^+^Mac1^+^Gr1^+^ myeloid cells from undifferentiated ESCs that were differentiated for 21 days “in vitro” under myeloid culture conditions. (C) CD45^+^Mac1^+^Gr1^+^ cells were FACS purified and then subjected to morphological analyses by May-Grünwald-Giemsa-staining. (D and E) Development of osteoclasts from undifferentiated ESCs that were differentiated for 16 days “in vitro” under osteocalst culture conditions. TRAP^+^ osteoclasts were detected from WT, Runx1^−/−^, and Runx1 expressing Tal-1^−/−^, and Runx1^−/−^ ESCs under the light microscope. (E) Addition of either c-fms antibody or osteoclast inhibiting factor (OCIF) to the culture blocked the development of osteoclasts from WT as well as Runx1 expressing Tal-1^−/−^ ESCs. Bars represent mean ± SD. SD: standard deviation, OP9: stromal cell line, ST2: stromal cell line, VitD_3_: VitaminD_3_, TRAP: tartrate resistance acid phophatase, c-fms: Colony stimulating factor 1 receptor.

Runx1 directly activates the expression of the transcription factor PU.1, which in turn, is required for the normal differentiation of myeloid cells (notably macrophages and osteoclasts) and B lymphocytes [39]–[41]. PU.1 expression is not detectable under Runx1- as well as Tal-1-deficient conditions of ESC differentiation (Fig. 4 A). This is consistent with our findings that no CD45^+^ cells were induced from Tal-1**^−/−^** ESCs that could be differentiated into either granulocytes (Gr1^+^), macrophages (Mac1^+^), or osteoclasts (TRAP^+^) under myeloid culture conditions (Fig. 1 A). Therefore, we tested whether retrovirally transduced Runx1 expression in Tal-1**^−/−^** cells induce the development of myeloid cells and osteoclasts.

When ESCs were kept under differentiating culture conditions “in vitro” in the presence of SCF, WT and also Runx1-expressing Tal-1**^−/−^** ESCs, but not Tal-1**^−/−^** ESCs were found now to express PU.1, though at lower levels (Fig. 4 A). In addition the “in vitro” differentiated WT- and Runx1-expressing cell cultures contained c-fms-expressing cells (Fig. 4 A) that were CD45^+^c-fms^+^ cells in FACS analyses (not shown). Such CD45^+^c-fms^+^ cells were not found in differentiating Tal-1**^−/−^** cell cultures. These results show that Runx1-expressing Tal-1**^−/−^** cells have the potential to differentiate to myeloid cells, although only in low numbers, compared with WT cells. Furthermore, after 21 days of culture, more mature, Mac1^+^ and Gr1^+^ macrophages and granulocytes were detected (Fig. 4 B). In a representative experiment 7.5×10^3^ undifferentiated WT ESCs at the start of the culture developed 5.6×10^4^ CD45^+^ cells under myeloid culture conditions (Fig. 1 A), of which 3.4×10^3^ became Mac1^+^Gr1^+^ on day 21 of the culture. In contrast, from Runx1-reconstituted Tal-1**^−/−^** ESCs only 5% (1.9×10^2^) of the cells became CD45^+^Mac1^+^Gr1^+^ cells under these culture conditions compared to WT ESCs (Fig. 4 B), and from Runx1-reconstituted Runx1**^−/−^** ESCs only 7% (2.4×10^2^) CD45^+^Mac1^+^Gr1^+^ cells developed. CD45^+^Mac1^+^Gr1^+^ cells were FACS purified and then subjected to morphological analyses by May-Grünwald-Giemsa-staining. Thereby, monocytes/macrophages and granulocytes which show the typical morphology in the nucleus were detected from differentiated WT ESCs (Fig. 4 C). Both, Runx1-expressing Runx1**^−/−^** and Tal-1**^−/−^** cells differentiated into morphological identifiable granulocytes and monocytes (Fig. 4 C). Tal-1**^−/−^** ESCs did not form any myeloid cells.

Runx1-expressing Tal-1**^−/−^** ESCs were furthermore cultured under conditions that allow the induction of osteoclastogenesis. This allowed their differentiation into multinucleated TRAP^+^ cells (Fig. 4 D and E). However, compared with WT cells only approximately 1/20 of the numbers of osteoclasts were formed. Tal-1**^−/−^** cells did not develop such osteoclasts. Again, also Runx1-expressing Runx1**^−/−^** ESCs were allowed to differentiate into multinucleated TRAP^+^ cells, this time with higher numbers than from untransduced Runx1**^−/−^** ESCs (Fig. 4 D and E). M-CSF and RANKL are known to be critical for osteoclastogenesis [26]. Therefore, an antagonistic anti-c-fms antibody, or a decoy receptor of RANKL, osteoprotegerin was added to the osteoclast-directed differentiating ESC cultures. Both factors inhibited osteoclastogenesis from WT and Runx1-reconstituted Tal-1**^−/−^** ESCs (Fig. 4 E). These results demonstrate that osteoclasts developed from Runx1-reconstituted Tal-1**^−/−^** ESCs show a comparable dependency on the M-CSF and RANKL signaling pathways, as do WT cells.

### Erythropoiesis from Runx1-expressing Tal-1^−/−^ ESCs

**Figure 5:**
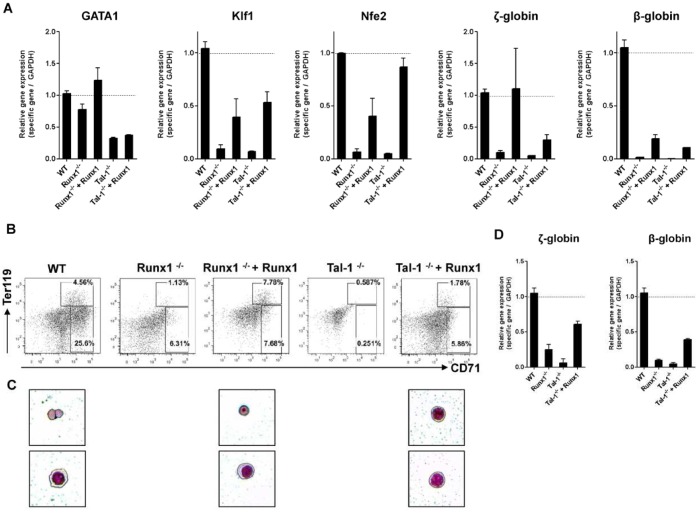
Development of primitive and definitive erythrocytes from Runx1-expressing Tal-1^−/−^ and Runx1^−/−^ ESCs. (A) qRT–PCR analyses were performed from differentiated WT, Runx1^−/−^, Tal-1^−/−^, and Runx1-expressing Tal-1^−/−^, and Runx1^−/−^ ESCs on day 8 of the culture out of 3 independent experiments. Bars represent mean ± SD. Development of (B) CD71^+^Ter119^+^ erythrocytes that were differentiated from undifferentiated cells. CD71^+^Ter119^+^ erythrocytes were FACS sorted and (C) then subjected to morphological analyses by May-Grünwald-Giemsa-staining and (D) qRT–PCR analyses were performed. FACS blots are representative examples of differentiated ESCs. OP9: stromal cell line, SCF: stem cell factor, rhEpo: murine rhecombinant erythropoietin, SD: standard deviation.

To determine whether erythropoiesis can be rescued by the ectopic expression of Runx1 in Tal-1**^−/−^** cells, ESC differentiated for 5 days were further cultured under erythroid conditions until day 8 (Fig. 1 C). Then, mRNA was probed by qRT-PCR for erythroid lineage-related genes, both of primitive as well as definitive erythropoiesis. β-globin and ζ-globin, GATA1, Klf1, and Nfe2, could be detected not only in mRNA of WT cells, but also in Runx1-reconstituted Tal-1**^−/−^** cells as well as in Runx1-reconstituted Runx1**^−/−^** ESCs (Fig. 5 A). These results are expected since it is known that Runx1**^−/−^** ESCs do not form definitive erythrocytes [10]. Additionally, primitive erythrocytes formed from Runx1**^−/−^** cells show an altered morphology and an altered expression profile for GATA1 and Klf1 [42]. Therefore, Runx1 plays a role not only in definitive but also in primitive erythropoiesis.

In our attempts to characterize the developed cells by the expression of erythroid surface markers, we found cells that had differentiated not only from WT-, but also from Runx1-reconstituted Tal-1**^−/−^** ESCs as well as from Runx1-reconstituted Runx1**^−/−^** ESCs to be Ter119^+^CD71^+^ (Fig. 5 B). These Ter119^+^CD71^+^ cells were FACS purified and then subjected to morphological analyses by May-Grünwald-Giemsa-staining. We could detect enucleated erythrocytes (definitive type) only from differentiated WT ESCs and from Runx1-reconstituted Runx1**^−/−^** cells, but not from Runx1-reconstituted Tal-1**^−/−^** cells (Fig. 5 C). Runx1-reconstituted Tal-1**^−/−^** cells did develop only into nucleated cells with the morphology of erythroblasts (Fig. 5 C).

To determine the ratio of primitive versus definitive erythrocytes in differentiated WT- and Runx1-expressing Tal-1**^−/−^** cells, we enriched the Ter119^+^CD71^+^ cells with FACS and performed qRT-PCR analyses for ζ-globin (primitive globin) and β-globin (definitive globin). The expression profiles indicate that inside of the Ter119^+^CD71^+^ cell fraction comparable levels of ζ-globin expression can be identified in both cell lines. However, in comparison to WT cells, β-globin expression was reduced in Runx1-expressing Tal-1**^−/−^** cells (Fig. 5 D).

We conclude from these results that retrovirally transduced Runx1 expression rescues primitive erythropoiesis more effectively than definitive erythropoiesis.

### Lymphopoiesis from Runx1-expressing Tal-1^−/−^ ESCs

**Figure 6:**
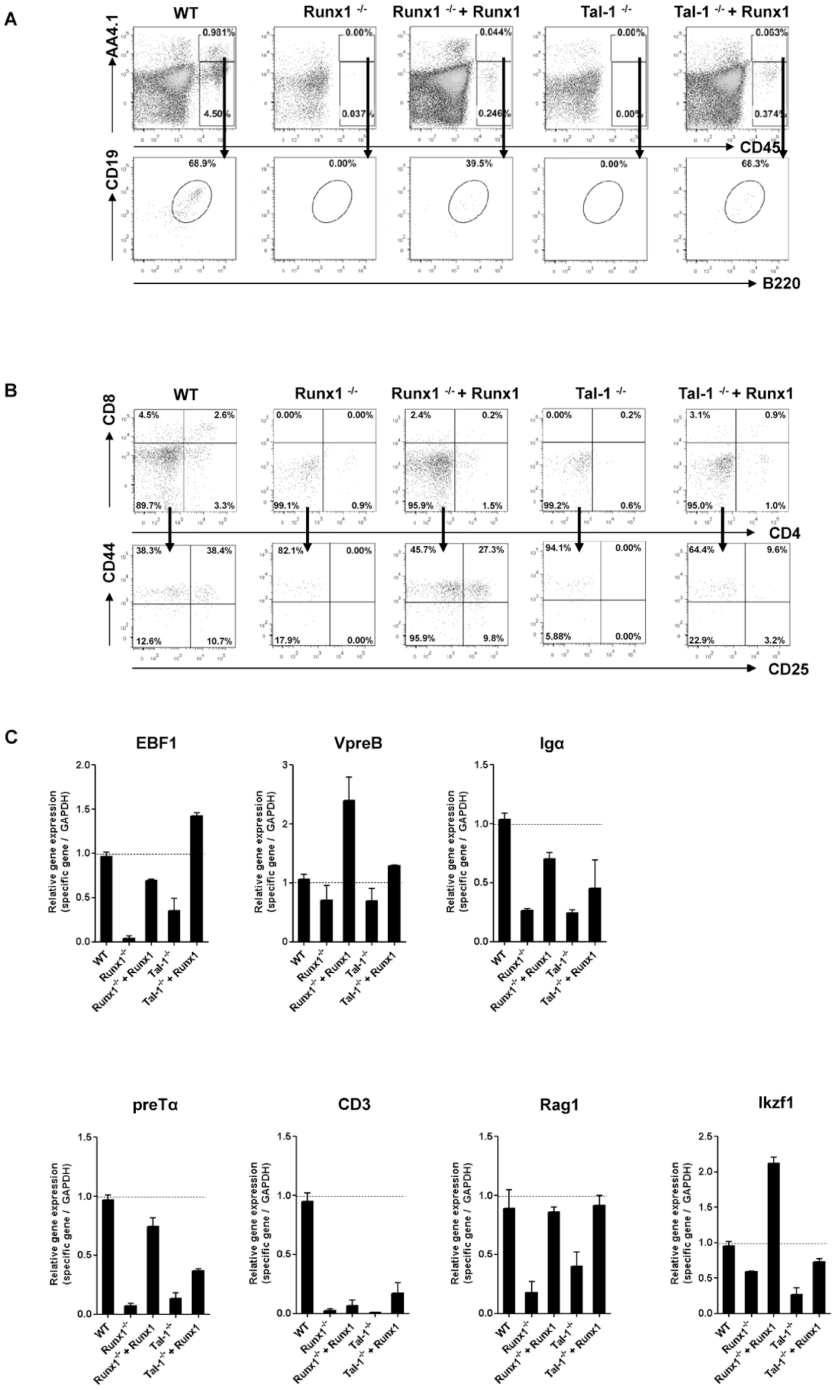
Development of lymphoid cells from Runx1-expressing Tal-1^−/−^ and Runx1^−/−^ ESCs. (A) For the development of AA4.1^+^CD19^+^B220^low^ B lymphoid cells from day 10 on IL-7 were added to the culture. (B) For the development of CD4^+^ and/or CD8^+^ T cells from day 10 on IL-7 were added to the culture and stromal cell layer were changed to DL1-OP9. FACS blots are representative examples of differentiated ESCs. (C) qRT–PCR analyses were performed from differentiated WT, Runx1^−/−^, Tal-1^−/−^, and Runx1-expressing Tal-1^−/−^, and Runx1^−/−^ ESCs on day 8 of the culture out of 3 independent experiments. Bars represent mean ± SD. OP9: stromal cell line, DL1: Delta-like 1, SCF: stem cell factor, IL: interleukin, Flt3-L: Fms-like tyrosine kinase 3 ligand; SD: standard deviation, CD: cluster of differentiation.

To further characterize Runx1-reconstituted Tal-1**^−/−^** cells, we used “in vitro” culture systems that allow the development of B and T lymphoid cells from ESCs. Thus, WT ESCs could be differentiated into preB cells, 68% (7.5×10^3^ cells) identified as CD19^+^B220^low^AA4.1^+^ (Fig. 6 A) and preT cells expressing CD4 (3.3% = 5.1×10^4^) and CD8 (4.5% = 6.4×10^4^ cells) (Fig. 6 B). Eight days differentiated WT ESCs expressed low levels of the lymphoid specific genes EBF1 as well as VpreB, Igα, Rag1, Ikzf1, CD3, and preTα (Fig. 6 C).

Consistent with the decreasing numbers of differentiating Tal-1**^−/−^** as well as Runx1**^−/−^** cells after day 5 of culture, neither preB nor preT cells could be identified in differentiating cultures of Tal-1**^−/−^** cells and Runx1**^−/−^** cells (Fig. 6 A and B). In contrast, the ectopic expression of Runx1 under Tal-1-deficient conditions rescued lymphoid phenotypes, as 68.3% of the surviving cells could be identified as CD19^+^B220^low^AA4.1^+^ under B lymphoid differentiation conditions (Fig. 6 A). Furthermore, CD4^+^ or CD8^+^ cells were developed under T lymphoid differentiation conditions (Fig. 6 B). From Runx1-expressing Runx1**^−/−^** ESCs only a few of the surviving cells could be identified as CD19^+^B220^low^AA4.1^+^ (39.5%; Fig. 6 A). Development of preT cells was more efficient from Runx1-expressing Runx1**^−/−^** ESCs (Fig. 6 B). On the molecular level, on day 8 of the culture, the expression of lymphoid lineage-associated genes was found to be downregulated in differentiating Tal-1**^−/−^** and Runx1**^−/−^** ESCs (Fig. 6 C). However, ectopic expression of Runx1 in Tal-1**^−/−^** and Runx1**^−/−^** ESCs resulted in an increase of the expression of B cell related genes (EBF1, VpreB, Igα, and Rag1), and to a lower efficiency the expression of T cell related genes (CD3, preTα, and Ikzf1) (Fig. 6 C) when compared to untransduced Tal-1**^−/−^** and Runx1**^−/−^** cells.

## Discussion

Tal-1 has been shown to be required for the development of both primitive and definitive hematopoiesis [8], [11], [12]. In Tal-1**^−/−^** embryos blast-colony-forming cells (BL-CFC), often also called hemangioblasts [43]–[49], i.e. progenitor cells of vascular endothelial cells, vascular smooth muscle cells and hematopoietic lineage cells develop, but do not generate progeny of vascular endothelium and hematopoietic lineages [13]. Induced expression of Tal-1 in Tal-1**^−/−^** cells at this early embryonic progenitor cell stage was shown to rescue both primitive and definitive hematopoiesis, but induction of Tal-1 expression at a later embryonic stage of development was ineffective [50]. Once pHSCs have been formed, e.g. in conditionally loxP-Tal-1-defective mice, Cre-recombinase-induced deletion of Tal-1 was found no longer to impair pHSC engraftment, self-renewal and differentiation into fetal myeloid and lymphoid lineage cells [14]. Thus, two classes of hematopoietic ‘stem cell’ transcription factors have been distinguished: those controlling the pHSC generation, and others controlling pHSC functions [14].

Previous studies have shown that Runx1 expression is directly controlled by Tal-1 [15]–[17]. Runx1 expression induced by Tal-1 at the hemangioblast stage of embryonic development appears mandatory for the development of clonogenic hematopoietic progenitors, as the loxP-Cre-mediated reversion of a Runx1**^−/−^** locus restores the proper function of Runx1. If induced during embryogenesis at the transition from hemangioblasts to pHSCs, it results in the development and the subsequent differentiation of fetal myeloid and lymphoid lineage cells [21]. Again, once pHSCs have been formed, Runx1-deficiency minimally affects the functions of pHSCs [51].

In our study, we have initiated ectopic expression of Runx1 by retroviral transduction of “in vitro” differentiating Tal-1**^−/−^** or Runx1**^−/−^** ESCs near the Flk1^+^Tie2^+^ mesodermal stage of embryonic development [44], [52], i.e. at a time and stage where hemangioblasts develop in these cultures [23], [24]. Thereby, we attempted to influence the generation, not necessarily the subsequent function of hematopoietic progenitors and their erythroid, myeloid and lymphoid lineage cells. To our surprise, Runx1 rescued both primitive and definitive hematopoiesis not only in Runx1**^−/−^** differentiating ESCs, but also in Tal-1**^−/−^** ESCs.

Compared with the “in vitro” differentiation of WT ESCs only at 1 to 10% of the primitive and definitive hematopoietic lineage cells developed. This included erythroid, myeloid and lymphoid lineage cells, as well as colony-forming units, which we, in fact, could detect at low frequencies when compared to experiments done by Nakano et al. only from WT [24], but not from Runx1-reconstituted Tal-1**^−/−^** and Runx1**^−/−^** cells. However, since these efficiencies were equally low for the rescue of both Tal-1**^−/−^** and Runx1**^−/−^** cells, we suspect that retroviral transductions are not well-enough controllable to allow expression of the transduced gene (Runx1) at the same, proper levels in all transduced cells. In fact, different levels of expression of PU.1 [39] or of Pax5 [53] have been seen to influence the capacities of hematopoietic cells to develop to different stages of differentiation.

Furthermore, we do not want to over-interpret the results of our experiments to conclude that retroviral transduction of “in vitro” differentiating ESCs by Runx1 leads to normal primitive and definitive hematopoiesis. Moreover, since we could not detect enucleated erythrocytes in cultures of Runx1-reconstituted Tal-1**^−/−^** cells, it is also possible that the expression of Tal-1 would be still necessary for the development of fully mature definitive erythrocytes even in the presence of Runx1. Nevertheless, ectopic expression of Runx1 was sufficient to rescue erythropoiesis regarding globin expression and the surface expression of Ter119 and CD71.

However, we think that our results show that Runx1 overexpression leads not quantitatively in proper cell numbers, but qualitatively in proper cell stages of primitive and definitive hematopoeitic lineage cells. Hence, both Tal-1 and Runx1 are transcription factors, which generate pHSCs, and Runx1 can take the place at the hemangioblast stage of embryonic development to generate hematopoiesis in the absence of Tal-1.

Tal-1 and Runx1 have also been seen to be required at later stages of hematopoiesis. For example, Tal-1 is required for proper differentiation of erythroid and megakaryocytic cells [14], and Runx1 is required in B and T lymphocyte development [54]–[58]. Therefore, our experimental results define the requirements for Tal-1 and Runx1 in the embryonic development of pHSCs, but not in the development and functions of later stages of erythroid, myeloid and lymphoid cell lineages. They should prove useful in tests of the pHSC-generating activities of genes detected in comprehensive expression analyses, e.g. of genes controlled by Tal-1 and Runx1 (Fig. 2) [16], [17], [34], [59], [60]. Our “in vitro” transduction protocol of Tal-1**^−/−^** and Runx1**^−/−^** differentiating ESCs could also be used to detect differences in the activities of mutated forms of these genes that are found in acute leukemia, therapy-derived leukemia, myelodysplastic syndrome, and chronic myelomonocytic leukemia.
